# Latent CMV Infection Is Associated With Lower Influenza Virus-Specific Memory T-Cell Frequencies, but Not With an Impaired T-Cell Response to Acute Influenza Virus Infection

**DOI:** 10.3389/fimmu.2021.663664

**Published:** 2021-05-05

**Authors:** Sara P. H. van den Berg, Josien Lanfermeijer, Ronald H. J. Jacobi, Marion Hendriks, Martijn Vos, Roos van Schuijlenburg, Nening M. Nanlohy, José A. M. Borghans, Josine van Beek, Debbie van Baarle, Jelle de Wit

**Affiliations:** ^1^ Center for Infectious Disease Control, National Institute for Public Health and the Environment, Bilthoven, Netherlands; ^2^ Center for Translational Immunology, University Medical Center Utrecht, Utrecht, Netherlands

**Keywords:** influenza infection, T cell, immune response, ageing, cytomegalovirus infection

## Abstract

Latent infection with cytomegalovirus (CMV) is assumed to contribute to the age-associated decline of the immune system. CMV induces large changes in the T-cell pool and may thereby affect other immune responses. CMV is expected to impact especially older adults, who are already at higher risk of severe disease and hospitalization upon infections such as influenza virus (IAV) infection. Here, we investigated the impact of CMV infection on IAV-specific CD8^+^ T-cell frequencies in healthy individuals (n=96) and the response to IAV infection in older adults (n=72). IAV-specific memory T-cell frequencies were lower in healthy CMV^+^ older individuals compared to healthy CMV^-^ older individuals. Upon acute IAV infection, CMV serostatus or CMV-specific antibody levels were not negatively associated with IAV-specific T-cell frequencies, function, phenotype or T-cell receptor repertoire diversity. This suggests that specific T-cell responses upon acute IAV infection are not negatively affected by CMV. In addition, we found neither an association between CMV infection and inflammatory cytokine levels in serum during acute IAV infection nor between cytokine levels and the height of the IAV-specific T-cell response upon infection. Finally, CMV infection was not associated with increased severity of influenza-related symptoms. In fact, CMV infection was even associated with increased IAV-specific T-cell responses early upon acute IAV infection. In conclusion, although associated with lower frequencies of memory IAV-specific T cells in healthy individuals, CMV infection does not seem to hamper the induction of a proper T-cell response during acute IAV infection in older adults.

## Introduction

The worldwide population is ageing rapidly. With age, deleterious changes in the immune system arise, referred to as ‘immunosenescence’, which impair responses against infectious diseases and vaccinations (1, 2). Age-related changes of the immune system mainly occur in the T-cell pool, including an increase in the number of CD45RA^+^ memory T cells (3, 4) and decreased diversity of the T-cell receptor repertoire (5, 6). About 2 decades ago, latent infection with cytomegalovirus (CMV) was implicated as a possible driving force of these age-related changes (7–9). CMV seropositivity was identified as part of the so-called ‘immune risk profile’, predictive of early mortality in older adults (7, 10). Moreover, CMV seropositivity is the largest non-heritable factor influencing differences among humans in the immune profile (11). CMV is generally thought to establish this large effect by its frequent attempts to reactivate during life-long carriage, thereby gradually affecting the immune system (12). Since the 2000s, it is often hypothesized that CMV might impair the human immune response to a heterologous challenge (6, 12, 13), as was shown in mice (14–16).

With age, the risk for serious complications and hospitalization after influenza virus (IAV) infection increases (17). Vaccination is an important tool to prevent infection, however also the efficacy of influenza vaccination decreases with age (2, 18). The role of CMV infection on influenza vaccination efficacy has been well studied. These studies have yielded conflicting results as a negative effect of CMV (19–21), a positive effect (1) as well as no effect of CMV on the vaccine response have been reported (22, 23). A systematic review by our group including a meta-analysis showed no clear evidence for a negative effect of CMV infection on the antibody response to influenza vaccination (24).

Both CMV and ageing primarily affect the T-cell compartment (3, 4, 25–27). During IAV infection, an effect of CMV would therefore mainly be expected on the T-cell response. T cells play an important role in clearance of the IAV (28). These T-cell responses are predominantly specific for the internal viral proteins, such as matrix protein-1 (29), which are conserved among influenza strains that have undergone antigenic drift. Indeed, pre-existing and early IAV-specific T-cell responses are associated with lower disease severity of influenza (30, 31), while delayed T-cell responses to IAV are thought to induce prolonged inflammation and delayed viral clearance and recovery (31, 32). Upon activation, IAV-specific CD8^+^ T cells show increased expression of activation markers (33, 34), produce proinflammatory cytokines and kill virus-infected cells by releasing perforin and granzyme B (29, 35).

Whether CMV infection attenuates the immune response to IAV infection in older adults remains unclear. There are two major hypotheses explaining how CMV infection may affect the immune response to a heterologous virus. First, it has been proposed that large clonal expansions of terminally differentiated CMV-specific CD8^+^ T cells, which are a hallmark of CMV infection and which can take up to 30%-90% of the CD8^+^ T-cell memory pool (36–38), may fill the ‘immunological space’ (10, 13, 39). Thereby CMV infection may hamper the induction of other immune responses (10, 13, 39). IAV-specific T cells may thus be outcompeted by CMV-specific T cells in their competition for proliferation and survival factors (13). Secondly, it has been suggested that CMV is linked to ‘inflammaging’, the lingering low-graded level of inflammation occurring with ageing (40). The production of pro-inflammatory mediators has been shown to enable CMV reactivation (41, 42). Upregulation of TNF-α, IL-6 and CRP have been observed in CMV-infected individuals, as well as increased production of IL-10 (11, 43, 44). Especially the increase of IL-10 combined with a decrease of IFNγ has been associated with reduced cytolytic capacity of CD8^+^ T cells responsible for clearing IAV, which also fits with the observed lower levels of granzyme B (45–47). Importantly, even though CMV has been suggested to diminish the T-cell response to IAV, there is no clinical evidence of a direct link between CMV infection and the T-cell response against IAV infection in humans.

We had the unique opportunity to study the effect of CMV infection on IAV in humans in the in a relatively large cohort of natural influenza infected older adults. We first investigated the effect of CMV infection on the presence of IAV-specific memory T cells in healthy young and healthy older individuals. Next we assessed the effect of latent CMV infection on the IAV-specific T-cell response in older adults undergoing an acute IAV infection. Our data show that CMV^-^infection is associated with reduced frequencies of IAV-specific T cells in healthy older adults, whereas in healthy younger adults no association with CMV infection is observed. Nevertheless, CMV infection does not hamper the T-cell response to acute IAV infection in older adults.

## Materials and Methods

### Study Design

#### Healthy Young and Older Adults

Samples of healthy individuals covering a broad age range were combined from two cohorts. Samples of young adults (n=34), between 18 and 52 years of age, from unvaccinated controls or pre-vaccination participants were selected based on age and sex from a study carried out in 2009-2011 (the Pandemic influenza vaccination trial, Netherlands Trial Register NL1952) (48). The study was approved by the Central Committee on Research Involving Human Subjects of the Netherlands. Samples of older adults (N=65), ≥60 years of age, were control samples from a study carried out in 2014-2015 (Influenza-like-illness-3, Netherlands Trial Register NL4666) (Kaaijk et al., submitted). This study was approved by the acknowledged ethical committee METC Noord Holland. Both studies were carried out in accordance with the recommendations of Good Clinical Practice with written informed consent from all subjects, in accordance with the Declaration of Helsinki.

#### IAV A Infected Older Adults During IAV Infection

Laboratory-confirmed Influenza virus A infected older adults were selected from the same study as the healthy older adults. In this prospective observational study participants were monitored for influenza-like-illness (ILI) in the influenza season of 2014-2015 (NL4666, Kaaijk et al., submitted). Study design of the Influenza-like-illness-3 study was comparable to previous studies as described in Van Beek et al. (49). In short, participants were instructed about influenza-like-illness (ILI) symptoms according to the Dutch Pel criteria, defined by fever (≥37.8°C) with at least 1 other symptom of headache, muscle pain, sore throat, coughing, runny nose, or chest pain (50) and to report ILI as soon as possible after onset. Nasopharyngeal and oropharyngeal samples were obtained within 72 hours of reporting ILI by standard procedures (49). IAV infection was laboratory confirmed, and subtyped by PCR and sequencing in n=72 individuals by methods described previously (49). The 72 IAV confirmed patients were included in the current study. The H3N2 strain was detected in the majority of patients (n=64, of which n=20 clade 3C.3b, n=37 clade 3C.2a, n=7 not determined), and the H1N1 strain in the remaining individuals (n=8). Blood samples were collected within the first 72 hours of fever onset, and followed up after 2 weeks and 8 weeks.

### PBMC and Serum Isolation

Peripheral blood mononuclear cells were obtained by Lymphoprep (Progen) density gradient centrifugation from heparinized blood, according to the manufacturer’s instructions. PBMCs were frozen in 90% fetal calf serum and 10% dimethyl sulfoxide at -135°C until further use. Serum was isolated out of tubes with clot-activation factor and stored at -80°C until further use.

### Cytomegalovirus (CMV)-Specific Antibodies

Anti-CMV IgG antibody concentrations were measured either using a commercial ELISA (IBL international GMBH) according to manufacturer’s instructions or by an in-house-developed multiplex immunoassay (51), depending on the cohort. For healthy young adults, CMV-specific antibody levels were measured using a commercial ELISA. Recommended cutoffs of the commercial ELISA kit were followed. Participants with a CMV antibody level of ≥12 U/ml or higher were considered CMV^+^, those with a level of ≤8 U/ml were considered CMV^-^, and those with a level between 8 and 12 U/ml were considered inconclusive and hence excluded for further analysis. For older healthy adults and IAV-infected individuals, CMV-specific antibody levels were measured in serum by our in-house-developed multiplex immunoassay. Cutoff were based on previous calculations: Individuals with a CMV-specific antibody level of ≤4 arbitrary units/ml were considered to be CMV^-^ and individuals with an antibody level > 7.5 RU/ml were considered CMV^+^, and those with a level between 4 and 7.5 arbitrary units/ml were considered inconclusive and hence excluded from further analysis (52). To reduce inter-assay variation, all samples from the same individual were measured on the same plate.

### Antigen-Specific T Cells by Flow Cytometry

#### Healthy Individuals

HLA-A2 positive healthy individuals were selected based on availability from young and old healthy individuals for subsequent IAV-specific T-cell analysis, by staining PBMCs for expression of HLA-A2 with the HLA-A2(BB7.2)-V450 antibody (BD Bioscience). Of the HLA-A2 positive individuals, ± 4 million PBMC’s were stained using the HLA-class I dextramer for epitope GILG of the M1 protein of IAV (A*0201/GILGFVFTL-APC, Immudex) for 20 minutes at room temperature. Surface staining was performed for 30 minutes at 4°C with the following antibodies: Fixable Viability Staining-780 (BD bioscience), CD3 (SK7)-AF700(BD bioscience), CD8(RPA-T8)-BrilliantViolet510, CD45RO(UCHL1)-BrilliantViolet711, CD27(O323)-BrilliantViolet786, CCR7(150503)-BrilliantUV395 (BD bioscience), KLRG-1(13F12F2)-PE-Cy7 (eBioscience), PD-1(EH12.2H7)-PerCP Cy5.5, CD95(DX2)-BrilliantViolet421 (BD Biosciences), CD127(A019D5)-BrilliantViolet650, CD57(HCD57)-PE and CXCR3(G025H7)-PE-Dazzle. All antibodies were purchased from Biolegend, unless stated otherwise. Acquisition was performed on a LSRFortessaX20 and data analysis was performed using FlowJo (Treestar). tSNE-analyses were performed using Cytobank (www.cytobank.org) (53) with for every donor 10.000 CD8^+^ T cells. Donors with less than 10.000 CD8^+^ T cells were excluded from t-SNE analysis (n=3).

#### IAV-Infected Individuals

Of all HLA-A2 positive individuals undergoing IAV infection (n=36), both IAV-specific T cells and CMV-specific T cells were assessed within the first 72 hours 2 and 8 weeks after fever onset. PBMCs were stained using the HLA-A2 dextramer for the GILG epitope of the M1 protein of IAV (A*0201/GILGFVFTL-APC, Immudex) (± 8 mln PBMCs) (detected in n=17) and the HLA-A2 dextramer for the NLV epitope of the pp65protein of CMV (A*0201/NLVPMVATV)-APC (Immudex) (1 mln PBMCs) for 20 minutes at room temperature. Extracellular staining was performed for 30 minutes at 4°C with the following antibodies: Fixable Viability Staining-780 (BD bioscience), CD3 (SK7)-AF700(BD bioscience), CD8(RPA-T8)-BrilliantViolet510 (Biolegend), CD45RO(UCHL1)-BrilliantViolet711 (Biolegend), CD27(O323)-BrilliantViolet786 (Biolegend), CCR7(150503)-BrilliantUV395(BD bioscience), KLRG-1(13F12F2)-PE-Cy7 (eBioscience), CD127(A019D5)-BrilliantViolet650 (Biolegend), CD57(HCD57)-PE (Biolegend) and CXCR3(G025H7)-PE-Dazzle (Biolegend), CD38(HIT2)-PE-Dazzle (BD bioscience) and HLA-DR(TU39)-BrilliantUV737 (BD Bioscience). Analysis was performed on an LSRFortessaX20.

### IAV-Specific and CMV^-^Specific IFNγ T-Cell Response by ELISpot

Virus-specific T-cell responses were quantified using the IFNγ enzyme-linked immunospot (ELISPOT) assay at all three time points for those of which enough cells were available (respectively n=66, n=61, n=58 out of n=72). Briefly, 400.000 PBMCs were stimulated with a 15-mer peptide-pool with 11 amino acids overlap, covering the total influenza M1 protein (1 µg/ml) (JPT) and incubated for 18 hours at 37°C on 96-well membrane-bottomed plates (PVDF plate MSIPS4510, Millipore) coated with anti-IFNγ mAbs (Mabtech). If indicated, IFNγ responses were corrected for the percentage of T cells in lymphocytes based on flow cytometry data. For CMV responses, 100,000 PBMCs were stimulated with a 15-mer peptide-pool with 11 amino acids overlap, covering either the UL55 (1μg/ml) (JPT), the IE-1 (1μg/ml) (JPT), or the pp65 (1μg/ml) (JPT) CMV protein. The sum of the response to these three CMV peptide pools is presented in this study.

### Cytokine and Chemokine Levels in Serum

Cytokines and chemokine levels in serum were assessed for all IAV-infected individuals within 72 hours, and 2 and 8 weeks after fever onset. Levels were measured by bead-based multiplex LEGENDplex™ (BioLegend) according to the manufacturer’s instructions. The pro-inflammatory cytokines IL-6, IFNy, IL-10 and CRP were analyzed for this study. Stimulations were performed in duplicate. Analysis was performed on a Canto II flowcytometer. Data were analyzed *via* Legendplex V8.0 software (Biolegend). All data were transformed into averages of the logarithms of two measurements, and each data point was corrected by subtraction of the intra-assay averages to correct for batch effects.

### Isolation of IAV-Specific T-Cells for T-Cell Receptor Analysis

CD8^+^ T cells were isolated from PBMCs using a negative selection microbeads kit (Miltenyi Biotec). Next, CD8^+^ T cells were labeled at room temperature for 20 minutes with the A*0201/GILGFVFTL-APC dextramer (Immudex) (GILG). Subsequently surface staining was added with the following mAbs: CD3(UCHT1)-PerCP (Biolegend), CD4(OKT4)-BV510 (Biolegend) and CD8(RPA-T8)-FITC (Biolegend) and CD3^+^CD4^-^CD8^+^GILG^+^ cells were sorted by FACS Melody (BD) directly into RNAlater (Ambion Inc. Applied Biosystems) and stored at -80°C for subsequent TCRβ clonotype analysis.

### Preparing TCRbeta cDNA Libraries for High Throughput Sequencing

T-cell receptor analysis was performed as described previously (54), with minor modifications. Briefly, mRNA was isolated with the RNA microkit (Qiagen) according to manufacturer’s protocol. Isolated mRNA was used for cDNA synthesis with 5’RACE template switch technology to introduce a universal primer binding site, and unique molecular identifiers (UMI’s) were added at the 5’ end of the cDNA molecules using the SMARTScribe Reverse Transcriptase (TaKaRa). cDNA synthesis was followed by an AMPure XP bead-based cleanup (Beckman Coulter). Purified cDNA molecules were amplified in two subsequent PCR steps using the Q5^®^ High-Fidelity DNA Polymerase (New England BioLabs), with an AMPure XP bead-based cleanup in between. PCR products were size selected on gel and purified using the Nucleospin PCR cleanup kit (Machery-Nagel). The PCR products were sequenced *via* Illumina MiSeq paired end using 2x250 bp sequencing.

### Analysis of TCRbeta Clonotype Analysis

Raw sequencing data was processed using the 12nt UMIs to correct for amplification biases and error-correction of reads. RTCR (55) was used to identify both the UMI sequence and clonotype information from the reads. Because of the relatively small number of cells per sample, additional filtering steps were followed to minimize cross-sample contamination and biases introduced by errors in the UMI sequence. Sequences were only accepted if their UMI was observed in at least 40 sequencing reads. Sequences with identical UMIs in multiple samples were removed if they did not occur in at least 1000 sequencing reads and also if their absolute frequency was lower than 10% of the maximum frequency in the other sample. UMIs were clustered within each sample within a Hamming Distance of 3.

### Severity of Symptoms Assessment

Symptom assessment was performed by self-reporting by the participants. As soon as a fever occurred, participants reported the already experienced symptoms up until then and subsequently reported the last day of each symptom until full recovery up until a maximum of timepoint three (8 weeks after start of fever). This way, the presence and duration (start date and end date) of the following symptoms were collected: fever (≥37.8°C), cough, sore throat, runny nose, headache, pain while breathing and muscle pain. Symptoms of an IAV-infected individual up until ten days before onset of fever were considered to be IAV infection related and included in the analysis in this paper. The duration of each symptom in days was normalized by transformation of these values to Z-values, e.g. the number of standard deviations by which the value of the score of an individual is above or below the mean value. Finally, to assess the overall severity of symptoms during IAV infection, the average of the Z-values of the seven symptoms was calculated.

### Statistical Analysis

Differences between groups (for example CMV^-^ compared to CMV^+^) were assessed using Mann-Whitney *U* test, and comparisons within the same individuals (for example to compare time points in response upon influenza virus infection) with the Wilcoxon singed-rank test. Differences between groups in categorical variables were tested by chi-square test and corrected for multiple testing if applicable and indicated in figure legends. Correlations were tested with Spearman’s rank correlation coefficient. For all analyses p values < 0.05 were considered statistically significant. Data were analyzed using GraphPad Prism 8.3 and SPSS statistics 22 for Windows (SPSS Inc., Chicago, IL, USA).

## Results

### Characteristics of Study Population

Healthy individuals were on average 59.2 years old (range 21-82 year) (n=99). They were categorized into young (21-52 years old) (n=34) and old (>60 years old) (n=65) individuals, of whom respectively 55.9% and 58.5% were CMV-infected (**Table 1**). No significant differences in age or sex were observed between CMV^-^ and CMV^+^ individuals (**Table 1**). In addition to healthy individuals, older adults (average 69.9 years, range 60-88 years) with confirmed IAV infection were included in this study (n=72). Also for the IAV-infected older adults, no significant differences in age or sex were observed between CMV^+^ and CMV^-^ individuals (**Table 1**). The majority of individuals were infected with the H3N2 strain (n=64/72), while some were infected with the H1N1 strain of influenza (n=8/72). All individuals in the H1N1 infected group turned out to be CMV^-^.

**Table 1:** Characteristics of the study population.

Healthy young adults				
	Total (n=34)	CMV- (n=15)	CMV+ (n=19)	Statistics
Age (mean ± SD)	35.9 ± 10.3	35.3 ± 10.8	36.4 ± 10.1	ns
Sex (% women)	61.8%	53.3%	68.4%	ns
CMV-serostatus (CMV+)	55.9%	.	.	.
***Healthy older adults***				
	***Total (n=65)***	***CMV- (n=25)***	***CMV+ (n=37)***	***Statistics***
Age (mean ± SD)	71.7 ± 6.6	70.9 ± 6.8	72.5 ± 6.5	ns
Sex (% women)	38.5%	33.3%	44.7%	ns
CMV-serostatus (CMV+)	58.5%	.	.	.
***Influenza virus infected older adults***				
	***Total (n=72)***	***CMV- (n=35)***	***CMV+ (n=37)***	***Statistics***
Age (mean ± SD)	69.9 ± 6.1	69.2 ± 5.3	70.4 ± 6.9	ns
Sex (% women)	41.7%	37.1%	45.9%	ns
CMV-serostatus (CMV+)	51.4%	.	.	.
Influenza virus strain (%H1N1)	11.1%	22.9%	0.0%	P=0.002

Differences were tested with Mann-Whitney U test for continues variables and chi-square test for categorical variables.

ns stands for non significant.

### CMV Induces an Increase in Senescence-Associated Markers in the T-Cell Pool in Older Adults

**Figure 1:**
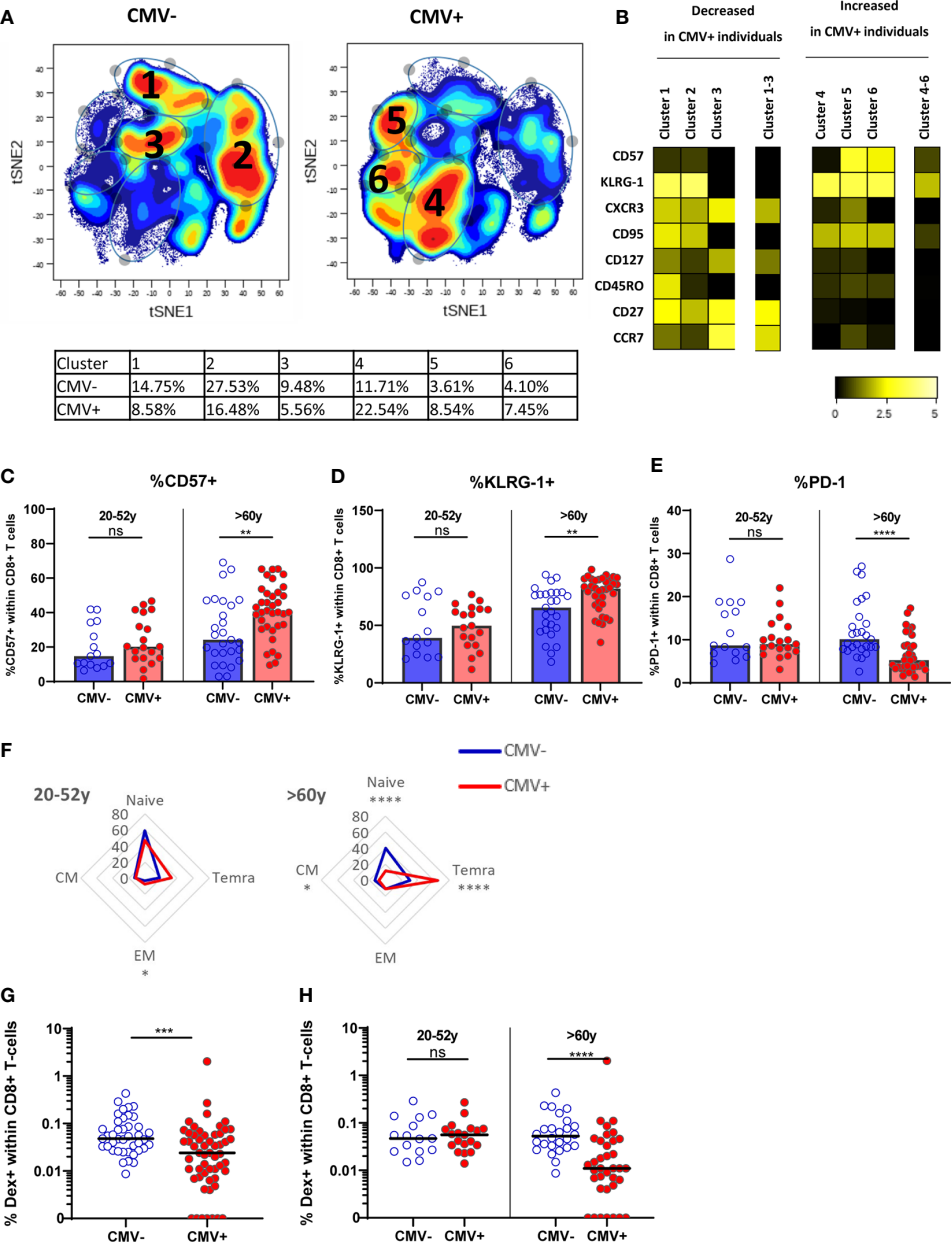
The effect of CMV on memory CD8^+^ T cells and IAV-specific CD8^+^ T cells in older adults. **(A)** t-SNE analysis of total CD8^+^ T cells based on MFI of CD57, KLRG-1, CXCR3, CD95, CD127, CD45RO, CD27 and CCR7 in CMV^-^ and CMV^+^ individuals (total of n = 96). **(B)** Heatmap of expression of markers of t-SNE clusters. Differences between CMV^-^ and CMV^+^ individuals is tested in **Supplementary Figure 1A**. **(C–E)** Percentage of CD57^+^ **(C)**, KLRG-1^+^ **(D)** or PD-1^+^ **(E)** CD8^+^ T cells in young and old CMV^-^ and CMV^+^ individuals. **(F)** Distribution of CD8^+^ T cells over naive and memory subpopulation in CMV^-^ and CMV^+^ individuals categorized in young (left panel) and old (right panel) adults. **(G)** Percentage of IAV-specific CD8^+^ T cells in all CMV^-^ and CMV^+^ individuals. **(H)** Percentage of IAV-specific CD8^+^ T cells in CMV^-^ and CMV^+^ individuals categorized in young and old individuals. Median is presented in each figure. Differences between groups were compared by Mann Whitney U test. **P < 0.01, ***P < 0.001, ****P < 0.0001. ns, non significant.

We assessed the effect of latent CMV infection on the CD8^+^ T-cell pool of all healthy individuals, by performing a cluster analysis (tSNE) based on memory T-cell markers CD27, CCR7, CD95, CD45RO, and CXCR3, and senescence-associated T-cell markers CD57 and KLRG-1, known to be altered in CMV infection (56). Cluster analysis conformed the large differences between the CMV^-^ (n=40) and CMV^+^ (n=56) group (**Figure 1A**), and six different clusters were identified. Clusters 1-3 containing non-senescent CD27^high^CCR7^high^ CD57^low^ cells were predominantly present in CMV^-^ individuals (**Figure 1B** and **Supplementary Figure 1A**). In contrast, clusters 4-6 containing the more differentiated cells expressing KLRG-1^high^CD57^medium^ were more pronounced in CMV^+^ individuals (**Figure 1B** and **Supplementary Figure 1A**). We next performed more detailed analysis on the expression of senescence-associated markers within the two age groups: young adults (20-52 years) and older adults (older than 60 years). CMV infection was associated with significantly increased expression of CD57 and KLRG-1 among older, but not in young, adults (**Figures 1C, D**). Likewise, expression of PD-1 was only significantly reduced in CMV^+^ individuals (as compared to CMV^-^) in the older group, and not in the younger group (**Figure 1E**). In line with this, CMV infection was associated with significantly increased frequencies of T_EMRA_ cells specifically in older individuals (**Figure 1F**). Together, this indicates that CMV infection establishes large changes in the CD8^+^ T-cell pool by inducing terminally differentiated and senescent T cells in older adults.

### Frequency of IAV-Specific T-Cells Is Decreased in CMV^+^ Individuals, but Only in Older Adults

To investigate the hypothesis that CMV infection may negatively influence the immune response to other pathogens by outcompeting other antigen-specific T cells, the frequency of IAV-specific T cells in healthy individuals was determined using HLA-A2 dextramers containing the matrix protein-1 GILG-epitope. The frequency of IAV-specific T cells was significantly lower in CMV^+^ compared to CMV^-^ individuals (P=0.0005) (**Figure 1G**). Importantly, this lower percentage of IAV-specific T cells was solely explained by a lower frequency in the older group, where some donors even had no detectable HLA-A2 GILG-specific T cells. Among the young adults no differences in the frequencies of IAV-specific T cells between CMV^-^ and CMV^+^ individuals were observed (**Figure 1H**). Thus, CMV infection results in lower frequencies of IAV-specific memory T cells, but only in older adults.

### Characterization of the T-Cell Response During IAV Infection in Older Adults

**Figure 2:**
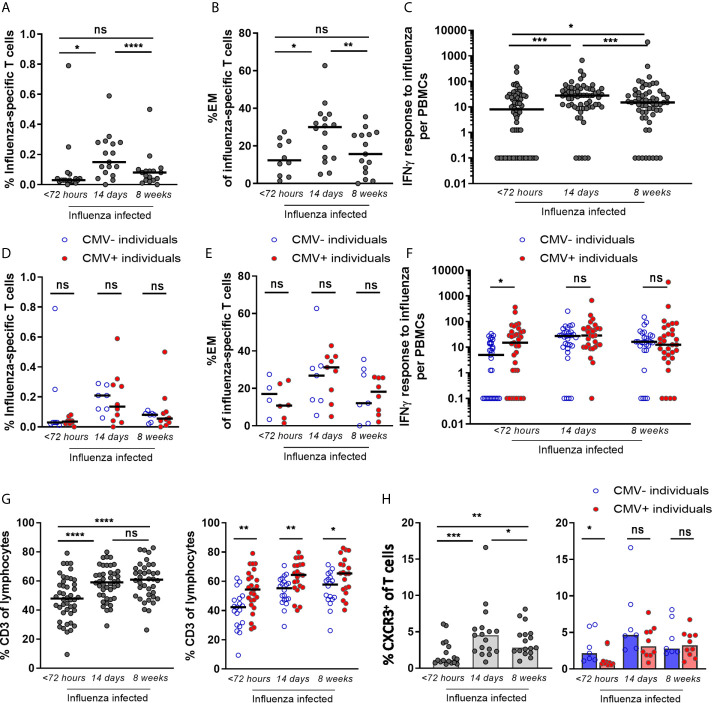
IAV-specific CD8^+^ T-cell response after infection shows no impairment by CMV seropositivity, but enhancement of the early IAV-specific CD8^+^ T-cell response. **(A)** Percentage of IAV-specific CD8^+^ T cells upon influenza infection. Dextramer for matrix protein-1 GILG-epitope was used **(B)** The percentage effector memory (EM) cells of IAV-specific CD8^+^ T cells upon influenza infection. All HLA-A2-positive influenza-infected individuals (n =36) were stained for dextramers; IAV-specific T cells were detected at least at one time point in 17 individuals. **(C)** IAV-specific IFNγ T-cell response upon influenza infection. **(D)** Percentage of IAV-specific T cells upon influenza infection for CMV^-^ and CMV^+^ individuals. **(E)** The percentage effector memory (EM) cells of IAV-specific T cells upon influenza virus infection for CMV^+^ and CMV^-^ individuals. **(F)** IAV-specific IFNγ T-cell response upon influenza infection for CMV^-^ and CMV^+^ individuals. **(G)** Frequencies of CD3^+^ T cells in the blood of IAV-infected individuals at < 72 hours, 2 weeks and 8 weeks after infection (left panel) and in CMV^-^ and CMV^+^ individuals (right panel). **(H)** Percentage of CXCR3^+^ within total CD8^+^ T cells upon infection (left panel) and in CMV^-^ and CMV^+^ individuals (right panel). Wilcoxon test was used to compare T-cell responses of individuals in time and Mann-Whitney U test was used to compare CMV^-^ and CMV^+^ individuals. Correlation between CMV-specific immune responses and IFNγ response to influenza virus was tested by Spearman correlation. T-cell IFNγ responses are presented per 1 x 10^6^ PBMCs. *P < 0.05, **P < 0.01, ***P < 0.001, ****P < 0.0001. ns, non significant.

To investigate the effect of CMV infection on the T-cell response during IAV infection, we characterized the T-cell response to IAV infection during infection in older adults (N=72), i.e. within 72 hours after start of fever, and 2 and 8 weeks later. The frequency of IAV-specific CD8^+^ T cells was determined dextramer staining for the HLA-A2 GILG-epitope. IAV-specific CD8^+^ T-cell frequencies were increased upon IAV infection at the 2 week time point compared to <72 hours after fever onset (median 0.03% to 0.15% of total CD8^+^ T cells respectively), after which the response contracted (8 weeks post infection, median of 0.08/total CD8^+^ T cells) (**Figure 2A**). The increase in IAV-specific CD8^+^ T-cell frequencies was mainly explained by the expansion of IAV-specific effector memory T cells (T_EM_) (**Figure 2B**), while the frequencies in the other subsets (i.e. naïve (T_N_), central memory (T_CM_) and T_EMRA_) did not increase (data not shown). IFNγ responses after *in vitro* stimulation of PBMCs with peptide pools covering the influenza matrix-protein-1 revealed similar results (**Figure 2C**).

This was true for both H3N2 influenza-infected and H1N1 influenza-infected individuals, and no difference in increase in the IFNγ response between the two strains was observed (data not shown). When the IAV-specific IFNγ response was calculated as percentage of CD3^+^ T cells instead of PBMCs, the IAV-specific IFNγ response also tended to be higher in response to IAV infection (2 weeks) compared to steady state (8 weeks), albeit not significant (p=0.059) (**Supplementary Figure 2A** left panel).

### T-Cell Response to IAV Infection in Older Adults Is Not Impaired by CMV Infection

To investigate whether the T-cell response during IAV infection (N=72) is influenced by CMV infection, we analyzed CMV^+^ and CMV^-^ individuals separately. No significant association of CMV^-^seropositivity on IAV-specific CD8^+^ T-cell percentages or on the percentage of T_EM_ cells among the IAV-specific T cells was observed (**Figures 2D, E**). Likewise, IAV-specific IFNγ responses were not negatively associated with CMV seropositivity (**Figure 2F**) or with the level of CMV-specific antibodies in CMV^+^ individuals at any of the time points (**Supplementary Figure 2B**). Surprisingly, in the acute phase (<72 hours after fever onset), CMV^+^ individuals even showed a significantly higher IAV-specific IFNγ T-cell response than CMV^-^ individuals (P=0.013) (**Figure 2F**). Also when corrected for the percentage of T cells among all lymphocytes, the same trend was observed (**Supplementary Figure 2A** right panel). This higher IAV-specific IFNγ T-cell response in CMV^+^ individuals could not be explained by the moment of sampling within the 72 hours after fever onset (data not shown).

A possible explanation for the higher IAV-specific IFNγ T-cell response in CMV^+^ individuals might be related to differences in T-cell migration. We therefore also investigated the total frequency of T cells in the blood and their expression of activation and migration markers upon IAV infection in CMV^-^ and CMV^+^ individuals. The frequency of total CD3^+^ T cells in the blood of influenza-infected individuals was significantly lower early after infection compared to 2 or 8 weeks later (**Figure 2G** left panel). Comparing CMV^+^ individuals with CMV^-^ individuals, the CD3^+^ T-cell frequency was also consistently lower in CMV^-^ individuals (**Figure 2G** right panel). Next, the migration of CD3^+^ T cells outside the blood, early after influenza infection, was further assessed by comparing the relative decrease within CMV^-^ and CMV^+^ individuals. The drop in CD3+ T-cell frequency early after IAV infection was significantly larger in CMV^-^ than in CMV^+^ individuals (**Supplementary Figure 3A**). Thus, the higher IFNγ IAV-specific responses in blood in CMV^+^ individuals early after influenza virus infection (**Figure 2F**) may at least partly be explained by higher frequencies of total T cells in CMV^+^ as compared to CMV^-^ individuals. The higher frequencies of total T cells may be a result of less migration of T cells. Investigating migratory capacity of T cells, we indeed found that early after IAV infection CXCR3 expression by CD8^+^ T cells, which is associated with migratory capacity of these cells to the lungs, was lower in CMV^+^ individuals compared to CMV^-^ individuals (**Figure 2H**). These data suggest that T cells in CMV^+^ individuals may be less prone to be recruited to the lungs early after IAV infection and thereby accumulate in the blood leading to higher frequencies. However, on IAV-specific CD8^+^ T cells, only a small trend of increased CXCR3 expression on (p=0.11) was observed in CMV^-^ compared to CMV^+^ individuals (**Supplementary Figure 3B**) early after IAV infection. In addition, the activation status of IAV-specific T cells by the frequencies of HLA^-^DR^+^CD38^+^ or CD127^-^KLRG^-^1^+^ revealed no difference between CMV^-^ and CMV^+^ individuals. IAV-specific T cells from CMV^-^ and CMV^+^ individuals both showed increased expression of these activation markers early after IAV infection (<72 hours after start fever) as compared to 2 and 8 weeks later, irrespective of CMV serostatus. This pattern was not seen for CMV-specific T cells, which served as a control. This suggests that activation and rapid expansion of IAV-specific memory T cells early after infection occurs irrespective of CMV serostatus (**Supplementary Figure 3C**). Overall, these data suggest that migration of T cells to the lungs might play a role in the enhanced IAV-specific IFNγ response observed in CMV^+^ individuals early after IAV infection (**Figure 2F**) although evidence remains circumstantial.

### Large CMV^-^Specific T-Cell Responses Are Not Associated With Impaired IAV-Specific T-Cell Responses

**Figure 3:**
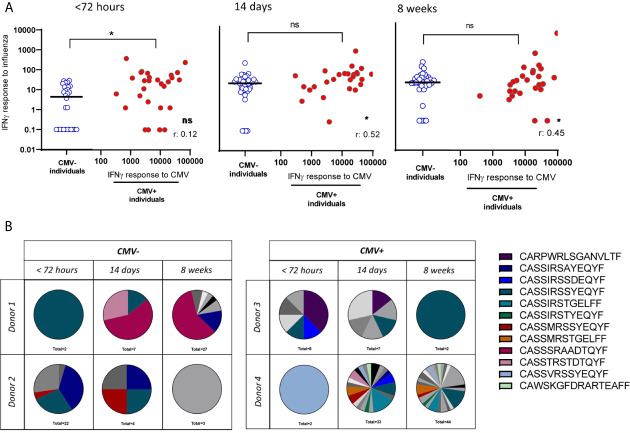
No reduced IAV-specific T-cell response in number of T cells or clonal diversity due to large expansions of CMV-specific T cells. **(A)** IAV-specific IFNγ T-cell response in CMV^-^ and CMV^+^ individuals differentiated on the CMV-specific T-cell response. IAV-specific IFNγ T-cell responses are depicted in blue open circles in scatter plot for CMV^-^ individuals and in red solid circles in correlation of CMV-specific IFNγ T-cell responses for CMV^+^ individuals. Differences were tested using Mann-Whitney to compare CMV^+^ and CMV^-^ individuals. Correlation between CMV-specific T-cell responses and IFNγ response to influenza was tested by Spearman correlation. T-cell IFNγ responses are presented per 1 x 10^6^ PBMCs. **(B)** T-cell repertoire of sorted HLA-A2-GILG–specific CD8^+^ T cells, detected by PCR of two CMV^-^ and two CMV^+^ individuals. Each pie-chart depicts the repertoire of a donor at a certain timepoint (< 72hours, 14 days or 8 weeks after infection). Colors represent shared CDR3 sequences between timepoints and donors. Grey scales depict unique CDR3 sequences. *P < 0.05. ns, non significant.

As CMV might only affect the immune system through competition for “limited immunological space” in individuals with large CMV-specific T-cell responses, we next investigated the association between CMV infection and the T-cell response to IAV by taking into account the magnitude of the IFNγ CMV-specific T-cell responses within CMV^+^ individuals. First, we assessed the IFNγ T-cell response to IAV infection. No negative correlation between expanded CMV-specific T-cell responses and T-cell response to IAV infection was observed at any of the three time points (**Figure 3A**). Surprisingly, we even observed a significant positive correlation between the height of the CMV-specific IFNγ T-cell response and the height of the IAV-specific IFNγ T-cell response at 2 and 8 weeks after fever onset (**Figure 3A**) (R: 0.52, p=0.016 and R:0.45, p=0.014 respectively. In addition, we further assessed the quality of the IAV-specific T-cell response by investigating TCR diversity of the IAV-specific T-cell response, by sorting the HLA-A2-GILG-specific T cells and sequencing the TCRβ chain. The clonotype distribution was analyzed over time after IAV infection in two CMV^-^ and two CMV^+^ individuals. In general it was observed that 89% of the different TCR sequences expressed the Vβ-19 segment, of which 74% consisted of the highly conserved -RS-motif (See **Supplementary Tables 1A, B** for the V and J segments). Interestingly, all donors showed a shift in dominance after IAV infection, however large heterogeneity was observed between the donors with some repertoires becoming more diverse and other less diverse over time. No indication for CMV-related differences were present, as no differences between the CMV^-^ and CMV^+^ individuals were observed. Based on these data no differences were observed in the TCR diversity of the IAV-specific T-cell repertoire between CMV^-^ and CMV^+^ individuals (**Figure 3B**). These lines of evidence do not support the competition between CMV-specific memory T cells and a proper IAV-specific T cells during IAV infection.

### Cytokine Levels in Serum of Influenza-Infected Individuals Are Not Affected by CMV and Not Associated With IAV-Specific T-Cell Responses

**Figure 4:**
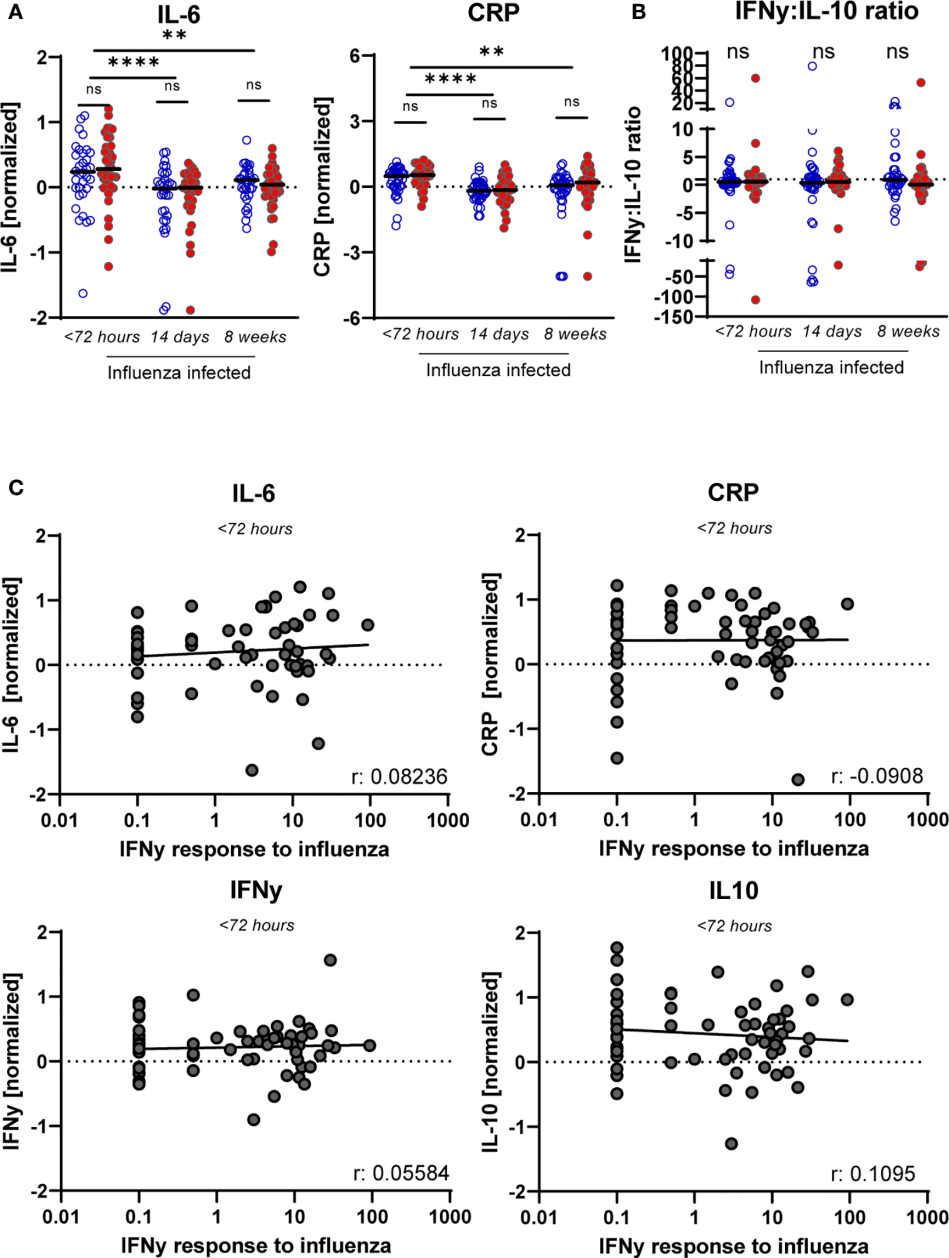
No sign of increased inflammation in CMV^+^ individuals based on cytokines levels in serum nor in relation to the IAV-specific T-cell response. **(A)** Serum levels of “inflammaging markers” CRP and IL-6 upon influenza infection for CMV^+^ and CMV^-^ individuals at < 72 hours after fever onset, and 2 and 8 weeks later. **(B)** The IFNγ:IL-10 ratio for CMV^-^ and CMV^+^ individuals upon influenza infection. **(C)** Magnitude of the IAV-specific IFNγ response at < 72 hours upon infection with influenza virus are not associated with the level of CRP, IL-6, IFNγ or IL-10 in serum. Serum levels of the cytokines were measured by multiplex assays and normalized based on subtracting the mean per plate. Differences were tested using unpaired T-test to compare CMV^-^ and CMV^+^ individuals. Correlations between IAV-specific IFNγ response and the different cytokines levels in serum were assessed by Spearman correlation. T-cell IFNγ responses are presented per 1 x 10^6^ PBMCs. **P < 0.01, ****P < 0.0001. ns, non significant.

As it is suggested that CMV is linked to “inflammaging”, we questioned whether pro-inflammatory mediators may be enhanced in CMV^+^ individuals during IAV-infection. We investigated the levels of pro-inflammatory cytokines in serum of CMV^+^ and CMV^-^ individuals, and their potential association with the IAV-specific T-cell response. At the early phase of IAV infection, the inflammation-associated factors IL-6 and CRP were elevated in serum compared to 2 and 8 weeks later (**Figure 4A**). No significant differences in IL-6 and CRP levels were observed between CMV^+^ and CMV^-^ individuals, also at the peak of the T-cell response (2 weeks later) and at steady state (week 8 after fever onset) (**Figure 4A**). We also measured the IFNγ:IL-10 ratio, as it was suggested that a shift in this ratio leads to a decline in IAV-specific T-cell responses with age and is associated with decreased protection against IAV (46, 57). No difference was observed (**Figure 4B**) in the IFNγ:IL-10 ratio between CMV^+^ and CMV^-^ individuals at any of the time points, even though both IFNγ and IL-10 levels were elevated at the earliest timepoint (**Supplementary Figure 4**). Although increased levels of the cytokines IL-6, CRP, IFNγ and IL-10 were observed in the acute phase of IAV infection, no significant association between these cytokine levels and the height of the IAV-specific T-cell response was observed <72 hours after start of fever (**Figure 4C**) or 2 and 8 weeks later (data not shown). Together, this suggests that CMV infection in older adults does not affect cytokine levels in serum upon IAV infection.

### Severity of Symptoms of IAV Virus Infection Is Not Increased by CMV

**Figure 5:**
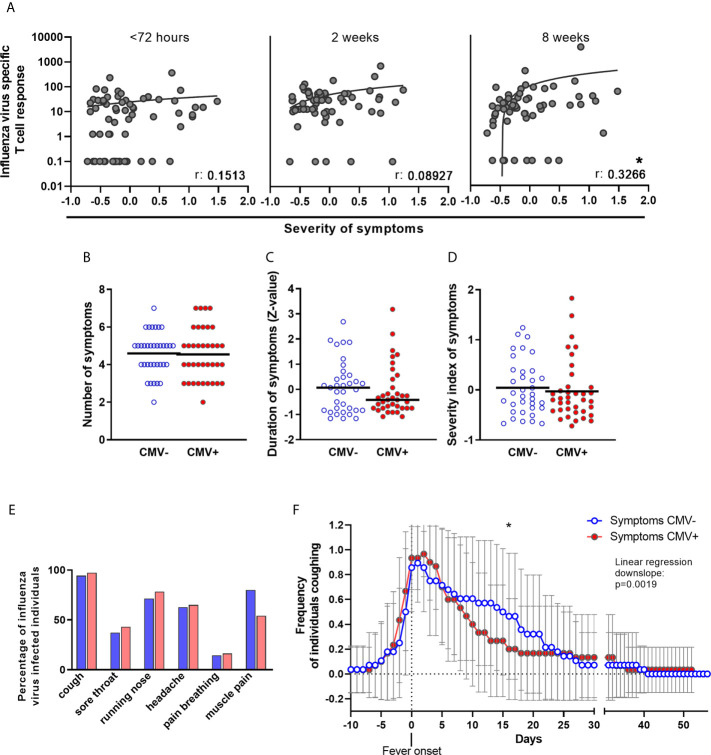
No negative effect of CMV-infection on number and duration of symptoms of influenza virus infection in older adults. **(A)** IAV-specific T-cell responses upon influenza virus infection at < 72 hours after fever onset, and 2 and 8 weeks later was associated with the severity of symptoms of influenza infection. Association was tested by Spearman correlation**. (B)** Number of symptoms during influenza virus infection in CMV^-^ and CMV^+^ individuals. Due to study design, participants had a minimal of two symptoms; fever (≥ 37.8°C) and at least 1 other symptom. Statistical differences between CMV^-^ and CMV^+^ were tested by χ-square test. **(C)** Duration of having symptoms, regardless which one and how many, of influenza virus infection in CMV^-^ and CMV^+^ individuals. Duration in days was calculated to Z-values. Difference between CMV^-^ and CMV^+^ individuals was tested by student-t test. **(D)** Severity of symptoms, taking along both duration and number of symptoms was assessed by taking the mean of Z-values of the six symptoms (cough, sore throat, runny nose, headache, pain while breathing or muscle pain). **(E)** Percentage of CMV^-^ and CMV^+^ individuals suffering from one of the six symptoms. Statistical differences between CMV^-^ and CMV^+^ were tested by χ-square test and corrected for multiple testing by Bonferroni correction. **(F)** Frequency of CMV^-^ and CMV^+^ individuals coughing during influenza virus infection. Difference between CMV^-^ and CMV^+^ individuals was tested by linear regression analysis on the downslope (starting at day of fever onset, day 0 on the x-axis) and comparison of the slope of CMV^-^ individuals and CMV^+^ individuals. *P < 0.05.

Since early T-cell responses during IAV infection play an important role in limiting disease severity, we also investigated whether the height of the IAV-specific T-cell response was associated with severity of symptoms of IAV infection. In IAV-infected older adults, the number and duration of the following symptoms was assessed: fever (≥37.8°C), cough, sore throat, runny nose, headache, pain while breathing and muscle pain. The severity of symptoms of IAV infection was positively associated with the height of the IAV-specific T-cell response at timepoint 3 (8 weeks after start of fever) (**Figure 5A**), but not within 72 hours or 2 weeks after start of fever. This association was mainly based on the number of symptoms, and not on their duration (**Supplementary Figure 5**). Thus, the IAV-specific T-cell response 8 weeks after IAV infection, and not the early T-cell response, was linked to the severity of symptoms of the IAV infection in this cohort.

We next investigated whether CMV infection was associated with more severe symptoms during IAV infection. No significant differences between CMV^-^ and CMV^+^ individuals were observed in the number of symptoms during IAV infection (**Figure 5B**), total duration of symptoms (**Figure 5C**), or severity of symptoms as assessed by a combination of duration and number of symptoms (**Figure 5D**). When investigating the IAV infection symptoms individually, CMV^+^ individuals tend to suffer less from muscle pain (**Figure 5E**) and although frequency of coughing was not different between CMV^+^ and CMV^-^, CMV^+^ individuals tended to suffer less long from coughing as a symptom compared to CMV^-^ individuals (data not shown). When the frequency of individuals coughing was plotted over time, we indeed found a indication for a faster decline in the frequency of coughing individuals amongst CMV^+^ individuals compared to CMV^-^ individuals (**Figure 5F**). Of note, both the increased frequency of muscle pain and the longer persistence of coughing among CMV^-^ individuals could not be ascribed to a difference in IAV strain infection between CMV^-^ and CMV^+^ individuals (data not shown). The severity of symptoms was also not associated with CMV^-^specific antibody levels (data not shown). Together, this suggests that CMV infection at least does not worsen the number and duration of IAV infection symptoms.

## Discussion

In this study we investigated the impact of CMV infection on the immune response to IAV. We found that CMV infection is associated with a more differentiated and senescent phenotype of CD8^+^ T cells. In healthy younger individuals, no difference in IAV-specific T-cell frequencies were observed, but, CMV^+^ older individuals had lower frequencies of IAV-specific memory CD8^+^ T cells compared to CMV^-^ older individuals. Nevertheless, the induction of an IAV-specific T-cell response during active IAV infection in older adults was not impaired. Also, severity of IAV-associated symptoms was not negatively affected by CMV infection. We did not find any evidence for a negative effect of CMV infection on the IAV immune response by ‘limited immunological space’ or by increased levels of pro-inflammatory mediators. In contrast, there seemed to be a positive association between CMV infection and the IAV-specific T-cell response early after IAV infection (<72 hours after fever onset).

To the best of our knowledge, we are the first to investigate the potential effect of CMV infection on the T-cell response to a heterologous infection in humans. A negative effect of CMV infection on the functioning of the immune system is often intuitively explained by competition between T cells for ‘limited immunological space’. Indeed, CMV infection has a profound impact on the composition of the overall CD8^+^ T-cell pool in healthy individuals, by increasing the number of highly differentiated memory cells, especially in older adults (25). We show that CMV infection leads to a decrease in the frequency of memory IAV-specific T cells in healthy older adults. Relatively low frequencies of IAV-specific T cells in older adults compared to younger adults have been reported before (58, 59) and are considered to be a key determinant of a diminished T-cell response in IAV infection (60). Note, that the observed frequencies and characteristics of the IAV-specific response are based on one HLA-A2 epitope (GILGFVFTL). Although this epitope is immunodominant and conserved between most influenza strains, it remains to be determined whether it reflects the T-cell total IAV-specific T-cell response.

Unfortunately, absolute T-cell numbers could not be investigated in this study, leaving the possibility that the decreased frequency of IAV-specific T cells merely reflected a relative increase in CMV-specific T cells in older adults. A decline in the frequency -and not in the number- of IAV-specific T cells may explain why decreased IAV-specific T-cell frequencies in healthy older adults do not seem to result in reduced T-cell responses upon acute influenza virus infection. This was also observed in the MCMV mouse model, were reduced frequencies were observed, but the absolute counts of CD8^+^ T cell against an heterologous virus infection were maintained in MCMV infected mice (61). Alternatively, the number of IAV-specific T cells may have been lower in CMV^+^ older adults. A similar effect has previously been reported for EBV-specific T cells (37). During IAV infection, we observed that IAV-specific T cells in CMV^+^ and CMV^-^ individuals responded equally well and no substantial changes in IAV-specific T-cell repertoire were observed in CMV^-^ and CMV^+^ individuals. Previously, to influenza vaccination, other studies reported impaired IAV-specific T-cell responses by CMV infection (20, 62, 63). In contrast, other groups showed a positive effect of CMV on the vaccine-induced IAV-specific T-cell response in humans (64). In addition, in mice, MCMV infection was shown to enhance the diversity of the T-cell repertoire against Listeria monocytogenes expressing OVA (65), whereas we found no differences in the repertoire diversity between CMV^-^ and CMV^+^ individuals. This could probably be explained by the small sample size of our repertoire analyses, or by the difference in epitope, as the HLA-A2-GILG specific T-cell repertoire is suggested to be more restricted compared to other antigen-specific repertoires (66). We also found that the magnitude of the CMV-specific T-cell response was positively associated with the magnitude of the IAV-specific T-cell response 2 weeks and 8 weeks after fever onset of IAV infection. Together, this suggests that CMV^+^ individuals have a sufficient amount of IAV-specific T cells able to respond to IAV infection.

Pre-existing T cells and timing of the IAV-specific CD8^+^ T-cell response are thought to play an important role in the reduction of severity of IAV-related symptoms (30, 31). An early T-cell response has been proposed to accelerate viral clearance, whereas a delayed and prolonged T-cell response may lead to high and prolonged levels of inflammation and increased severity of disease (31, 32). Unfortunately, pre-existing T-cell responses could not be investigated in this study. Our data suggest that CMV^+^ individuals have an increased early or accelerated IAV-specific T-cell response compared to CMV^-^ individuals. We speculate that this may lead to earlier viral clearance, and thereby might explain the faster recovery of coughing and decreased frequency of muscle pain. When we assessed severity of symptoms of symptoms, we found a significant positive correlation between the magnitude of the IAV-specific T-cell response and severity of symptoms 8 weeks after fever onset, and not early after fever onset or 2 weeks later. This suggests that a prolonged T-cell response, still present 8 weeks after fever onset, is increasing symptom severity of IAV infection.

As CMV infection was suggested to induce a more inflammatory environment (67), we also studied the potential association between CMV and inflammatory markers and how this might be related to IAV-specific T-cell responses. Although the levels of pro-inflammatory cytokines and chemokines measured here were significantly increased in the acute phase of IAV infection (<72 hours after fever onset), they were similar between CMV^+^ and CMV^-^ individuals. Since pro-inflammatory responses were similar between CMV^+^ and CMV^-^ individuals, it may (partly) explain the lack of difference in the severity of clinical symptoms between these groups. Furthermore, these levels did not seem to be associated with the IAV-specific T-cell response at any time point, although, we cannot rule out the possibility that other immune modulatory factors may play a role.

Most studies claiming a negative effect of CMV on an immune response to a heterologous challenge have been performed in mice. Several mouse studies have shown that only lifelong infection with MCMV leads to decreased immunity against heterologous infections (14–16). The magnitude of the effect of CMV infection might thus be linked to the duration of CMV infection and the experienced amount of viral reactivation in an infected host. We found no evidence for decreased immunity against a heterologous infection in humans infected with CMV. Also, high CMV-specific antibody levels were not associated with the height of the IAV-specific T-cell response. One of the reasons for the observed differences between mice and men, could be the order of infections by CMV and IAV. In mouse studies, mice are typically first infected with MCMV long before they are challenged with a heterologous acute infection. Many humans, in contrast, may have undergone their first IAV infection before they were infected with CMV, which may lead to the presence of IAV-specific memory T cells before the CMV-specific immune response is established. A potential harmful effect of CMV might be less pronounced in a host who already has a proper immune response against IAV. Furthermore, as mouse models of CMV are almost exclusively done in specific pathogen free mice, and humans are exposed to dozens of infections and triggers during life, it might be that the effect of CMV is magnified in mice. Even if CMV can modulate other immune responses, there is no substantial evidence that CMV impacts the function of the immune system by hampering immune responses against heterologous infections in humans.

The increased IFNγ T-cell response to IAV infection that we observed in CMV^+^ older individuals remains partially unexplained. Previously, enhanced influenza vaccine responses in humans and mice were explained by an increase in IFNγ in serum (1). However, we did not observe a difference in IFNγ levels in serum between CMV^+^ and CMV^-^ individuals in our study. However, an important difference between our study and the study by Furman et al. is the age of the individuals, as the latter observed this increase in IFNy levels mainly in younger adults. As the positive effect of CMV was observed in IAV IFNγ ELIspot assays, we cannot exclude the possibility that CMV infection may only affect CD4^+^ T cells, which may respond in the assay as well. Another explanation for the difference in the early IAV-specific T-cell response between CMV^-^ and CMV^+^ individuals could be related to the migratory capacity of the responding T cells, as we observed a trend towards enhanced CXCR3 expression of IAV-specific CD8^+^ T cells in CMV^-^ individuals. This may lead to early migration towards tissues such as the lungs. The effect of CMV on IAV-specific T-cell responses at the site of infection instead of the blood would be of great interest, and requires further research. Also, other factors like apoptosis or proliferation of the IAV-specific T cells may play a role in the difference in IAV-specific T-cell response between CMV^-^ and CMV^+^ individuals.

In conclusion, identification of the driving forces that induce age-related changes in the immune system is important to protect the growing population of older adults against infectious diseases. Especially IAV leads to more increased disease burden in older adults, e.g. severe symptoms and higher risk of complications, hospitalization and mortality. Our study shows that despite the lower frequency of IAV-specific memory T-cell responses in older adults, CMV infection does not seem to impair the T-cell response against acute IAV infection. This work supports the view that CMV acts, if anything, as an immune modulatory mediator, rather than having a true negative impact on the immune system.

## Data Availability Statement

Datasets are available on request: The raw data supporting the conclusions of this article will be made available by the authors, without undue reservation. 

## Ethics Statement

The studies involving human participants were reviewed and approved by ethical committee METC Noord Holland. The patients/participants provided their written informed consent to participate in this study.

## Author Contributions

The original idea for this study was from DV. SV performed the majority of experiments, gathered data and analysis. JL performed a large part of the experiments, gathered data and analysis. RJ, MH, MV, NN, and RV performed and evaluated experiments. JV designed the original study of IAV infected individuals. JD with help from JB and DV supervised the project. SV and JL prepared figures and wrote the manuscript with contributions and review from JD, DV, and JB. All authors contributed to the article and approved the submitted version.

## Funding

Funded by Dutch Ministry of Health Welfare and Sport.

## Conflict of Interest

The authors declare that the research was conducted in the absence of any commercial or financial relationships that could be construed as a potential conflict of interest.
