# Supplementary material for: Latent CMV Infection Is Associated With Lower Influenza Virus-Specific Memory T-Cell Frequencies, but Not With an Impaired T-Cell Response to Acute Influenza Virus Infection
Source: Front Immunol. 2021 May 5;12:663664. doi: 10.3389/fimmu.2021.663664 (PMC8131658; doi:10.3389/fimmu.2021.663664)
Supplement: Supplementary file 1 [file DataSheet_1.pdf]

Donor	Timepoint	Number of UMI's	CDR3β	Vβ	Jβ
**1**	<72h	2	CASSIRSSYEQYF	VB19	JB2-7
	14d	1	CASSIRSSYEQYF	VB19	JB2-7
		4	CASSSRAADTQYF	VB19	JB2-3
		2	CASSTRSTDTQYF	VB19	JB2-3
	8w	1	CASSARAAYEQYF	VB19	JB2-7
		1	CASSARGADTQYF	VB19	JB2-3
		1	CASSARSAYEQYF	VB19	JB2-7
		1	CASSFRAAYEQYF	VB19	JB2-7
		2	CASSIRGAYEQYF	VB19	JB2-7
		4	CASSIRSAYEQYF	VB19	JB2-7
		16	CASSSRAADTQYF	VB19	JB2-3
		1	CASSSRAAYEQYF	VB19	JB2-7
**2**	<72h	1	CASSIGTGEQFF	VB19	JB2-1
		8	CASSIRSAYEQYF	VB19	JB2-7
		6	CASSIRSSYEQYF	VB19	JB2-7
		1	CASSMRSSYEQYF	VB19	JB2-7
		6	CASSSFYLNEQFF	VB19	JB2-1
	14d	1	CASSIRSAYEQYF	VB19	JB2-7
		1	CASSIRSSYEQYF	VB19	JB2-7
		1	CASSMRSSYEQYF	VB19	JB2-7
		1	CASSQLAGWDEQYF	VB27	JB2-7
	8w	3	CASSYMSSGNTIYF	VB19	JB1-3

Supplemental table 1a. GILG+ T cell clones of CMV- individuals

Donor	Timepoint	Number of UMI's	CDR3β	Vβ	Jβ
3	<72h	3	CARPWRLSGANVLTF	VB24-1	JB2-6
		1	CASSIRSSDEQYF	VB19	JB2-7
		1	CASSIRSSYEQYF	VB19	JB2-7
		1	CASSSRSAETQYF	VB19	JB2-5
		1	CASSSRSSETQYF	VB19	JB2-3
		1	CASSSRSSGELFF	VB19	JB2-2
	14d	1	CARPWRLSGANVLTF	VB24-1	JB2-6
		1	CASSIIAGGQGEGQPQHF	VB19	JB1-5
		1	CASSIRSSYEQYF	VB19	JB2-7
		1	CASSSRSTDEQYF	VB19	JB2-3
		1	CASSTRSSGELFF	VB19	JB2-2
		2	CASTKGVYTQYF	VB12-4	JB2-3
	8w	2	CASSIRSSYEQYF	VB19	JB2-7
4	<72h	2	CASSVRSSYEQYF	VB19	JB2-7
	14d	2	CASSARSTGELFF	VB19	JB2-2
		1	CASSIRSADEQFF	VB19	JB2-1
		1	CASSIRSAGEQFF	VB19	JB2-1
		1	CASSIRSAYEQYF	VB19	JB2-7
		2	CASSIRSSDEQYF	VB19	JB2-7
		3	CASSIRSSYEQYF	VB19	JB2-7
		1	CASSIRSTDEQYF	VB19	JB2-3
		5	CASSIRSTGELFF	VB19	JB2-2
		1	CASSIRSTGEQFF	VB19	JB2-2
		1	CASSIRSTYEQYF	VB19	JB2-7
		1	CASSIRTSYEQYF	VB19	JB2-7
		1	CASSIRTTGELFF	VB19	JB2-2
		2	CASSMRASYEQYF	VB19	JB2-7
		2	CASSMRSTGELFF	VB19	JB2-2
		1	CASSNAGTIYEQYF	VB18	JB2-7
		1	CASSSRSSYEQYF	VB19	JB2-7
		2	CASSTRSTDTQYF	VB19	JB2-3
		1	CASSVRSAYEQYF	VB19	JB2-7
		1	CASSVRSSYEQYF	VB19	JB2-7
		1	CASTVSGVTYNEQFF	VB19	JB2-1
		1	CAWSKGFDRARTEAFF	VB30	JB1-1
	8w	1	CASRGTGDPNSPLHF	VB2	JB1-6
		1	CASSGRSTDTQYF	VB19	JB2-3
		1	CASSGRSTGELFF	VB19	JB2-2
		7	CASSHRSTGELFF	VB19	JB2-2
		1	CASSIGSFGYTF	VB19	JB1-2
		1	CASSIRSSVEQFF	VB19	JB2-1
		6	CASSIRSSYEQYF	VB19	JB2-7
		1	CASSIRSTDAQYF	VB19	JB2-3
		1	CASSIRSTDEQFF	VB19	JB2-1
		2	CASSIRSTDTQYF	VB19	JB2-3
		1	CASSIRSTETQYF	VB19	JB2-5
		3	CASSIRSTGELFF	VB19	JB2-2
		1	CASSIRSTYEQYF	VB19	JB2-7
		1	CASSLRTTDEQFF	VB19	JB2-1
		2	CASSLWTTDEQFF	VB19	JB2-1
		1	CASSMRSSYEQYF	VB19	JB2-7
		3	CASSMRSTGELFF	VB19	JB2-2
		1	CASSRRSTDEQYF	VB19	JB2-7
		1	CASSRRSTDTQYF	VB19	JB2-3
		2	CASSTRSTDEQYF	VB19	JB2-3
		1	CASSTRSTYEQYF	VB19	JB2-7
		1	CASSVRSSDEQYF	VB19	JB2-7
		1	CASSVRSSYEQYF	VB19	JB2-7
		1	CASSWRSSYEQYF	VB19	JB2-7
		1	CASSWRSTGELFF	VB19	JB2-2
		1	CAWSKGFDRARTEAFF	VB30	JB1-1

Supplemental table 1b. GILG+ T cell clones of CMV+ individuals

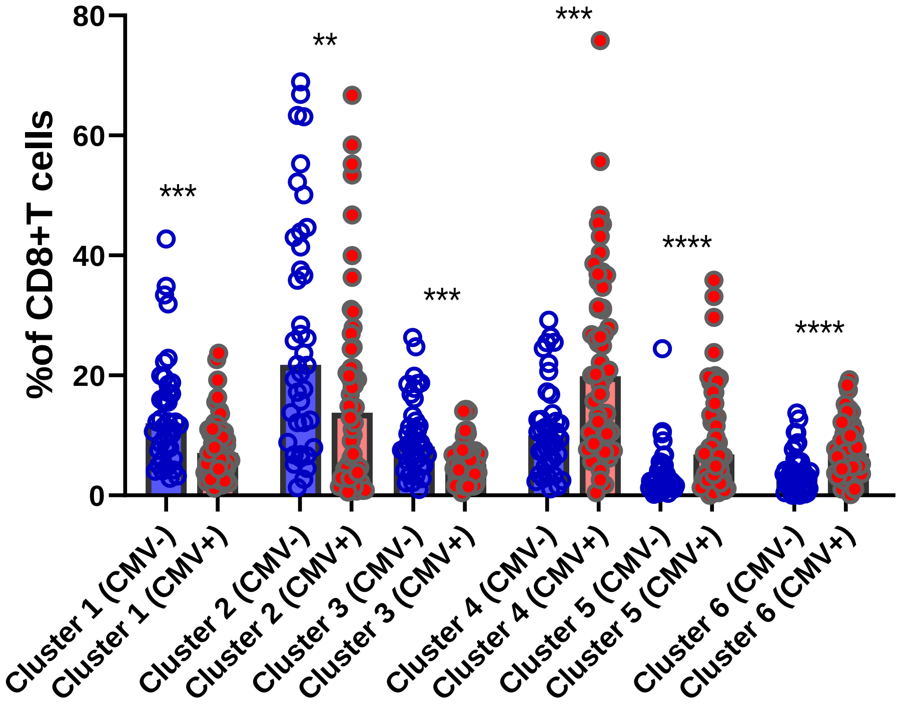

Supplementary figure 1. Percentage of CD8+ T-cells per t-SNE cluster in CMV$^-$ and CMV$^+$ individuals. Percentage of CD8$^+$ T cells per t-SNE cluster in CMV$^-$ (blue bars; N=40) and CMV$^+$ (red bars; N=57) individuals based on MFI of CD57, KLRG-1, CXCR3, CD95, CD127, CD45RO, CD27 and CCR7. Data shown as median. Differences between groups were compared by Mann Whitney. Stars indicate statistical differences, e.g. * P < 0.05, **P < 0.01, ***P < 0.001, ****P < 0.0001.

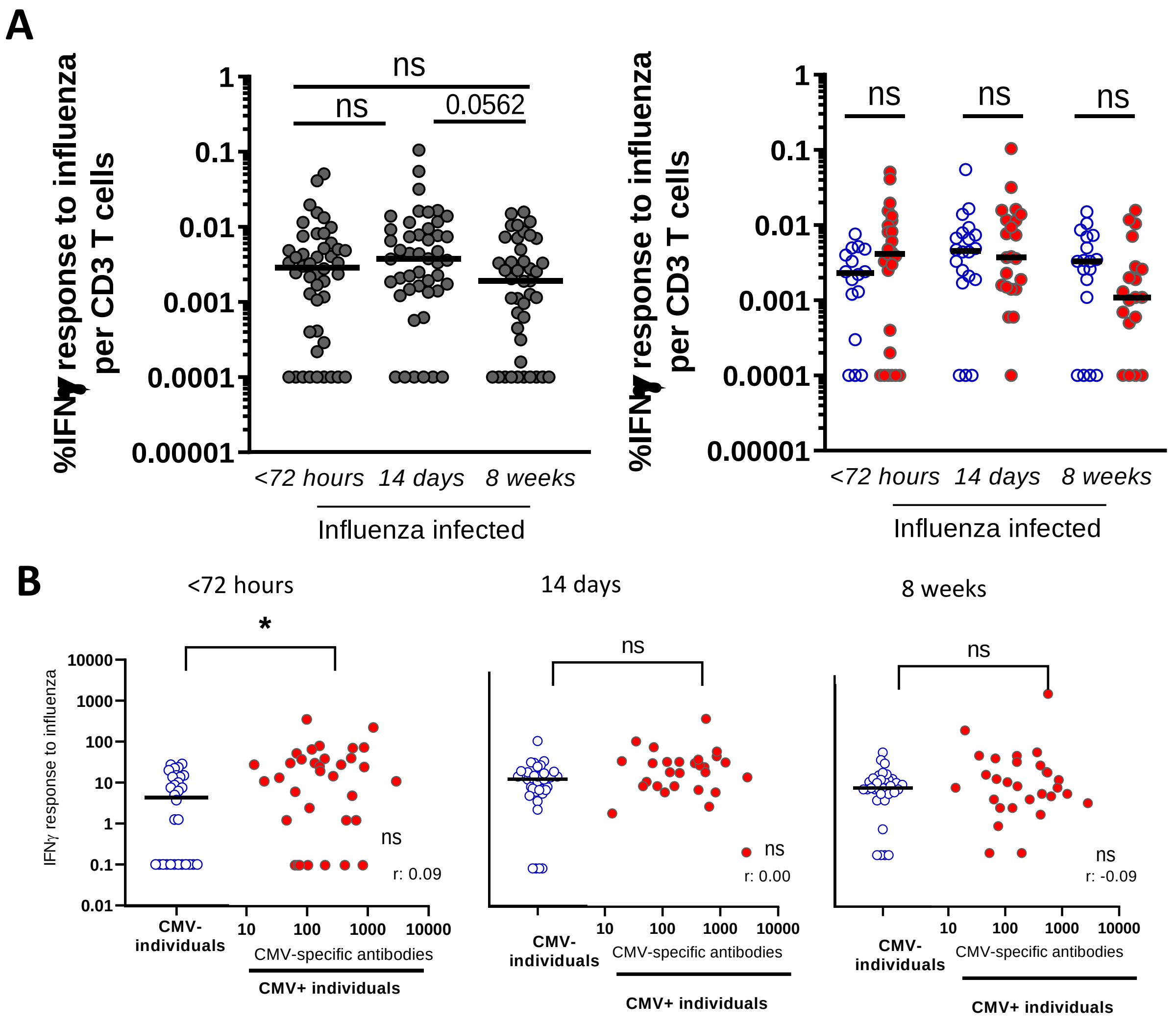

Supplementary figure 2. Influenza virus-specific IFNγ T-cell response in CMV$^-$ and CMV$^+$ individuals differentiated on the level of CMV-specific antibodies. A) All influenza virus-specific IFNy response at <72 hours, 2 weeks and 8 weeks after fever onset, depicted as percentage of total CD3 T cells (left panel), and compared between CMV$^-$ and CMV$^+$ individuals (right panel). **B)** Correlation between CMV-specific antibody levels and influenza virus-specific T-cell responses in CMV$^+$ individuals on the three time points after fever onset. Influenza-specific IFNγ T-cell responses are depicted in blue open circles in scatter plot for CMV$^-$ individuals and in red solid circles in correlation of CMV-specific IFNγ T-cell responses for CMV$^+$ individuals. Correlation was assessed by Spearman correlation.

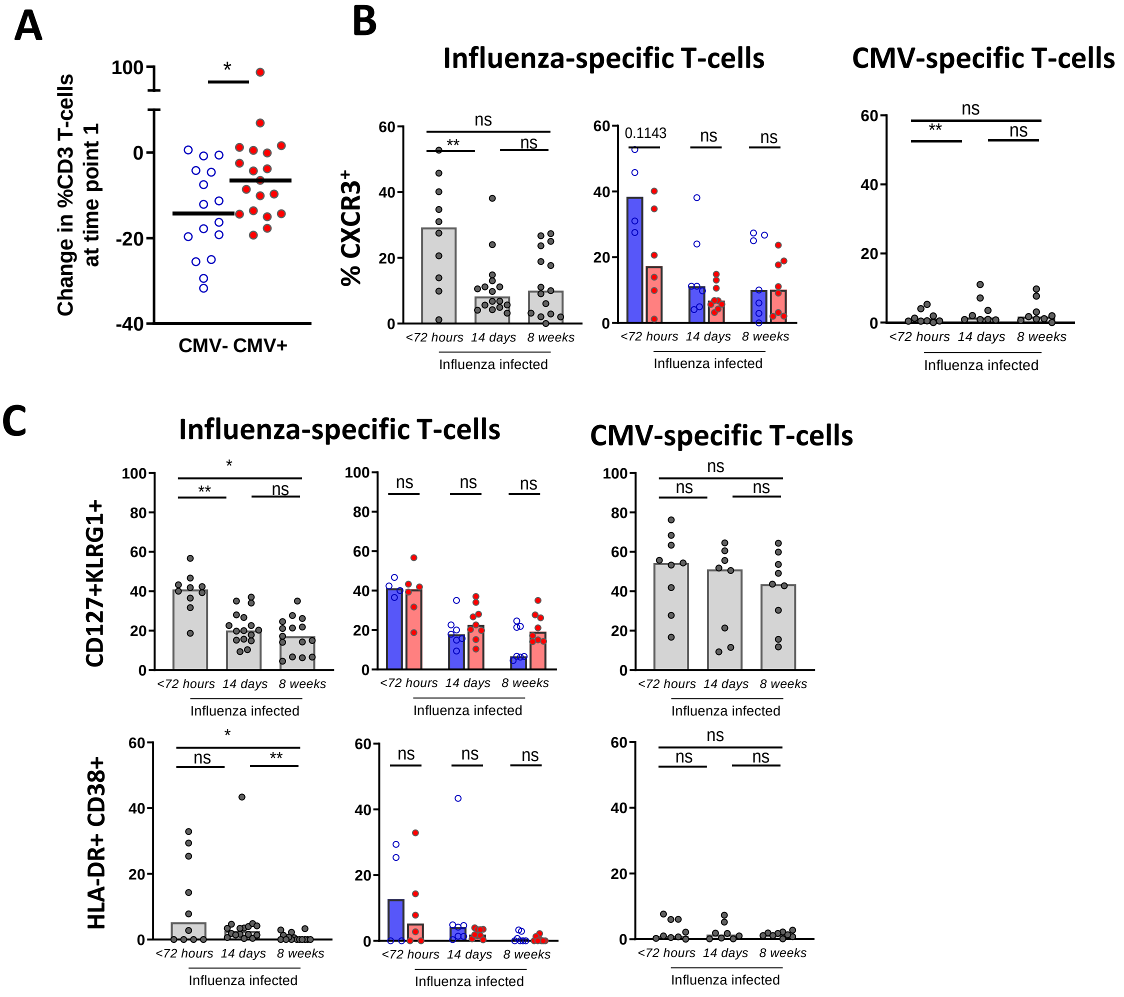

Supplementary figure 3. Expression of activation and migration markers in T cells during influenza virus infection. A) The change in percentage of $CD3^+$ T cells at time point 1 compared to time point 2 in CMV^- and CMV^+ individuals. **B)** $CXCR3^+$ influenza-virus specific $CD8^+$ T cells upon infection (left panel) and CMV^- and CMV^+ individuals (middle panel) and on CMV-specific $CD8^+$ T cells (right panel). **C)** The percentage of CD127-KLRG-1^+ and HLA-DR^+CD38^+ influenza virus-specific $CD8^+$ T cells upon influenza infection in all HLA-A2 positive individuals upon influenza infection (left panels), or comparing CMV^- (blue bars) and CMV^+ (red bars) (middle panels). Right panels shows $CD127^-$KLRG-1^+ and HLA-DR^+CD38^+ expression on CMV-specific $CD8^+$ T cells. Influenza virus-specific $CD8^+$ T cells were identified using a tetramer for matrix protein-1 GILG-epitope. CMV-specific $CD8^+$ T cells were identified using a tetramer for pp65 protein NLV-epitope. Wilcoxon test was used to compare time points. Differences between CMV^- and CMV^+ individuals were tested using Mann-Whitney U test.

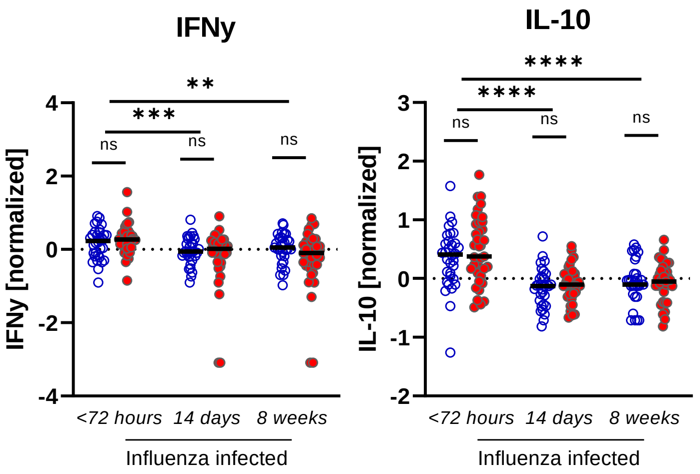

Supplementary Figure 4. Serum levels of IFNγ and IL-10 in CMV$^-$ and CMV$^+$ individuals after influenza virus-infection. Serum levels of IFNγ and IL-10 upon influenza infection for CMV$^+$ and CMV$^-$ individuals at <72hours after fever onset, and 2 and 8 weeks later. CMV$^-$ individuals are indicated with the blue open circles and the CMV$^+$ individuals in red solid circles. Serum levels of the cytokines were measured by multiplex assays and batch effects were normalized based by subtracting the mean per plate. Differences between CMV$^-$ and CMV$^+$ individuals were tested using unpaired T-test. Differences between time points were tested by Wilcoxon test.

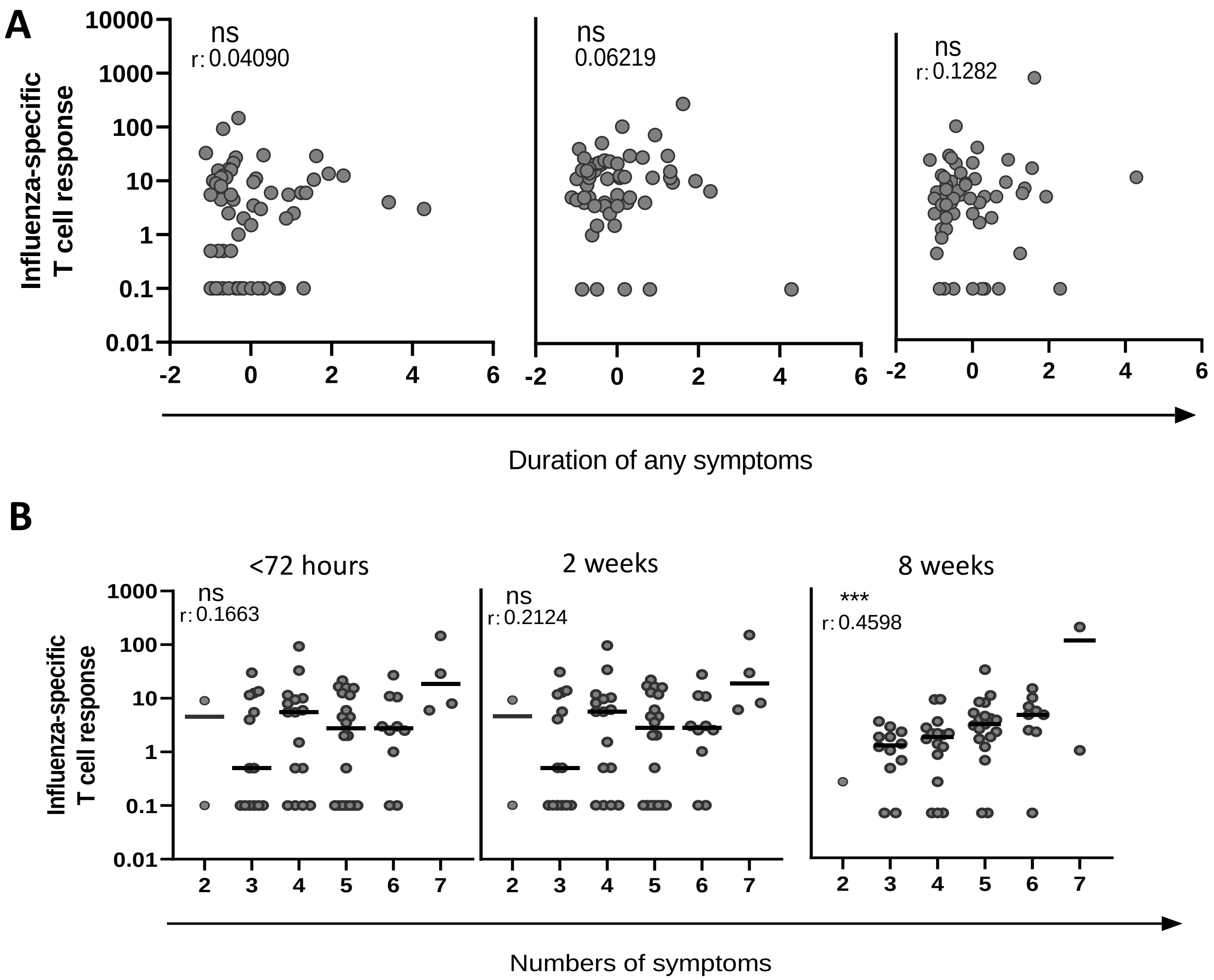

Supplementary Figure 5. Duration of symptoms is not linked to T-cell responses to influenza virus-infection. A) Association was tested for influenza-specific T-cell responses upon influenza virus infection at <72 hours after fever onset, and 2 and 8 weeks later with the duration of symptoms of influenza infection. **B)** Association was tested for influenza-specific T-cell responses upon influenza virus infection at <72 hours after fever onset, and 2 and 8 weeks later with the number of symptoms. Due to study design, participants had a minimal of two symptoms; fever (≥37.8 °C) and at least 1 other symptoms, either cough, sore throat, runny nose, headache, pain while breathing or muscle pain. Associations was tested by Spearman correlation.
